# Classroom experiments with artificial sweeteners: growing single crystals and simple calorimetry

**DOI:** 10.1107/S2056989022007617

**Published:** 2022-08-02

**Authors:** Johan Wouters, Luc Van Meervelt

**Affiliations:** a Chemistry Department, Université de Namur, rue de Bruxelles 61, B-5000 Namur, Belgium; b Chemistry Department, KU Leuven, Celestijnenlaan 200F, B-3001 Leuven, Belgium; University of Kentucky, USA

**Keywords:** crystal growth, erythritol, xylitol, calorimetry, *Mercury*, WebCSD

## Abstract

An easily accessible experimental set-up to grow large single crystals of two sweeteners readily available in supermarkets, erythritol and xylitol, is described. The crystallization of these compounds illustrates the principles of crystallization by evaporation. As these artificial sweeteners are also known to induce a cool sensation in the mouth, the crystal-growing experiment is complemented with a simple calorimetric set-up to demonstrate the endothermic nature of the dissolution of the selected sweeteners in a more qu­anti­tative approach by measuring the heat of dissolution (Δ*H*_diss_). The choice of these two sweeteners also allows use of the freely available WebCSD and *Mercury* tools to visualize their three-dimensional structure, crystal packing and powder diffractograms, and to deepen stereochemical concepts such as stereoisomers and *meso* compounds.

## Abstract 2

An easily accessible experimental set-up to grow large single crystals of two sweeteners readily available in supermarkets, erythritol and xylitol, is described. The crystallization of these compounds illustrates the principles of crystallization by evaporation·The crystal-growing experiment is complemented with a simple calorimetric set-up to demonstrate the endothermic nature of the dissolution of the selected sweeteners in a more qu­anti­tative approach by measuring the heat of dissolution.

## Introduction

Growing large single crystals is a fascinating experience for young and old. The success of several crystal growing competitions clearly proves this (Van Meervelt, 2014 ▸, 2017 ▸). The compound to be crystallized during such competitions should be readily available, at a reasonable price (often with the support of a sponsor) and should crystallize within a reasonable period (a few weeks). The substance must also be non-toxic. The solubility in water should be moderate to reduce the amount of substance to be used.

During different editions of the Belgian Crystal Growing Competition for example, various substances were crystallized: potassium alum (double sulfate of aluminium and potassium), borax, potassium di­hydrogen phosphate, copper sulfate, ammonium iron sulfate, ammonium magnesium sulfate, Seignette salt and even co-crystals of α-d-glucose and sodium chloride were recently grown.

In all cases, the substance comes from a chemical supplier, making experimentation by individual students more difficult. This can easily be solved by using substances available in the kitchen at home. Several artificial sweeteners are good candidates! Moreover, some of these show a cool sensation in the mouth, which can be linked to their endothermic heat of dissolution (Δ*H*_diss_). This enthalpy change associated with the dissolution of the sweetener in water can be measured by calorimetry.

An accessible experimental set-up to grow large single crystals of artificial sweeteners starting from easily available household products together with a simple calorimetric approach to determine their heat of dissolution is presented. At the same time, these experiments can be the starting point for the introduction of concepts such as crystal growth, stereochemistry and crystallographic databases.

## Artificial sweeteners

**Figure 1:**
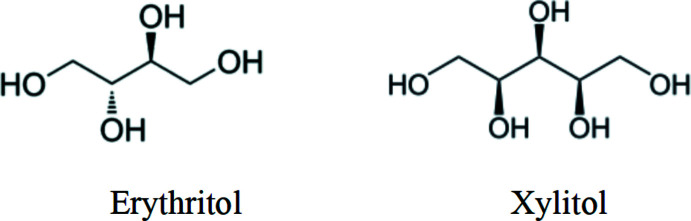
Structures of erythritol and xylitol.

Two sweeteners, erythritol and xylitol, were chosen for the crystallization and calorimetric experiments (Fig. 1 ▸). These two polyols are substitutes for sucrose, the classic table sugar. Both have a lower calorie intake and do not lead to dental caries problems.

Erythritol, C_4_H_10_O_4_, or (2*R*,3*S*)-butane-1,2,3,4-tetrol, is the *meso* (*R*,*S*) compound of butane-1,2,3,4-tetrol (diastereomer of l-threitol and d-threitol). This sweet polyol occurs naturally in some algae and lichens. It is also present in various fruits and fermented products.

Xylitol, C_5_H_12_O_5_, also known as birch sugar, can be produced from the bark of this tree or from xylan polymers present in biomass. It is also present in small amounts in many fruits and berries. The systematic name is 2,3,4,5-tetra­hydroxy­penta­nol or (2*R*,3*R*,4*S*)-pentane-1,2,3,4,5-penta­nol, the latter emphasizing the stereochemistry of the three chiral centers of the mol­ecule.

**Figure 2:**
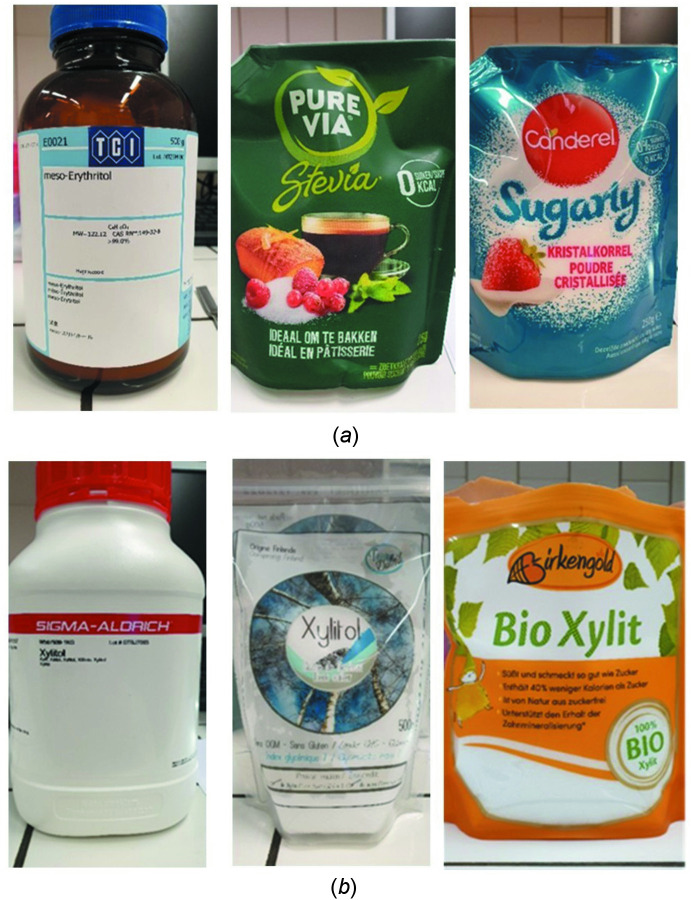
Commercial sources of (*a*) erythritol and (*b*) xylitol used.

Three commercial sources of erythritol were evaluated: TCI, Stevia and Canderel (Fig. 2 ▸ *a*). Similarly, three commercial sources of xylitol were used: Sigma, Xylitol from Bio-Cap and Bio-Xylit from Bio Planet (Fig. 2 ▸ *b*). While TCI and Sigma are specialized suppliers of laboratory reagents, the other sources are readily available in supermarkets in Belgium. The crystalline powders were used without further purification. Of course, there are other sources of these sweeteners, but they were not explored further.

**Figure 3:**
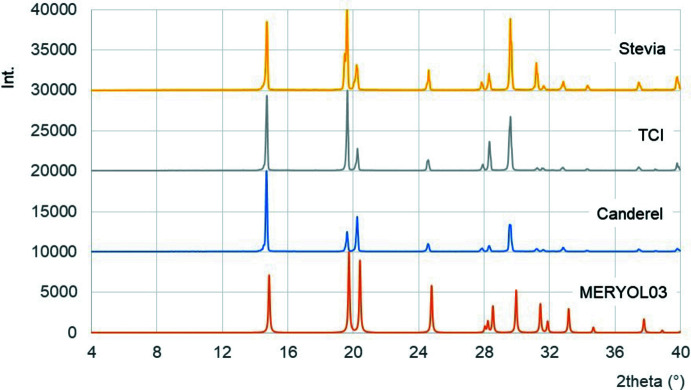
Comparison of the powder diffractograms [intensities (Int.) as a function of the diffraction angle 2θ] for erythritol from different sources. The reference diffractogram was simulated using *Mercury* (Macrae *et al.*, 2020 ▸) based on the crystallographic structure of MERYOL03 (Ceccarelli *et al.*, 1980 ▸).

X-ray diffraction analysis of the powders of the two polyols led to the conclusion that in both cases each powder corres­ponds to the same crystalline form as described in the literature (Fig. 3 ▸). The experimental diffractograms were compared with diffractograms simulated using *Mercury* (Macrae *et al.*, 2020 ▸) based on crystallographic data available in the Cambridge Structural Database (CSD, version 5.43, update of March 2022; Groom *et al.*, 2016 ▸; CSD refcodes XYLTOL01 and MERYOL03; Madsen *et al.*, 2003 ▸; Ceccarelli *et al.*, 1980 ▸). In this comparison, mainly the positions of the diffraction peaks, expressed in 2θ values, are used.

This result indicates that the commercial sweeteners used contain essentially the compound of inter­est. Any other components are either present in too low concentrations and/or are not crystalline and hence undetectable by X-ray diffraction. This approach illustrates the value of X-ray powder diffraction as a ‘barcode’ or ‘fingerprint’ of a crystalline solid, but requires equipment not available outside a specialized laboratory. Little influence of the source of the sweeteners was observed in the results, with all samples, for example, leading to the formation of single crystals. However, better results were obtained with reagents from laboratory suppliers, presumably due to a higher degree of purity.

## Growing single crystals

There are several methods of obtaining crystals, such as cooling a liquid (*e.g.* formation of ice cubes or snowflakes by freezing water), sublimation (*e.g.* growing crystals from iodine vapor), reaction between substances in the vapor phase (*e.g.* formation of ammonium chloride crystals by reaction of HCl_(g)_ and NH_3(g)_ vapors), precipitation reactions in solution (*e.g.* precipitation of silver chloride by reaction between Ag^+^_(aq)_ and Cl^−^_(aq)_ ions), or evaporation of solvents (*e.g.* recovery of table salt from seawater in salt marshes), to name only the most classic cases.

(Re)crystallization by evaporation of solvents relates to the solubility of a compound in the solvent and is closely related to the concept of saturation. In this regard, the influence of temperature on the solubility of a solute is also a parameter that can be used to obtain crystals. Cooling a saturated solution (for example obtained by dissolving when heated) should be added to the above methods. However, a distinction must be made between crystallization by cooling a pure liquid (*e.g.* solidification of water into ice) and crystallization by cooling a solution (*e.g.* crystallization of salt by cooling a salt solution). According to Le Chatelier’s principle, obtaining crystals by cooling a solution only applies in the case of solutes characterized by an endothermic heat of dissolution (Δ*H*_diss_ > 0).

A general protocol to grow large single crystals is available at the IUCr website under the heading ‘Outreach’, see https://www.iucr.org/outreach/crystal-growing-competition-2019/info-for-newcomers. A short movie ‘How to grow a single crystal’ is available at http://www.iycr2014.org/__data/iucr/iycr2014/video/SingleCrystal_DEF.mp4.

**Figure 4:**
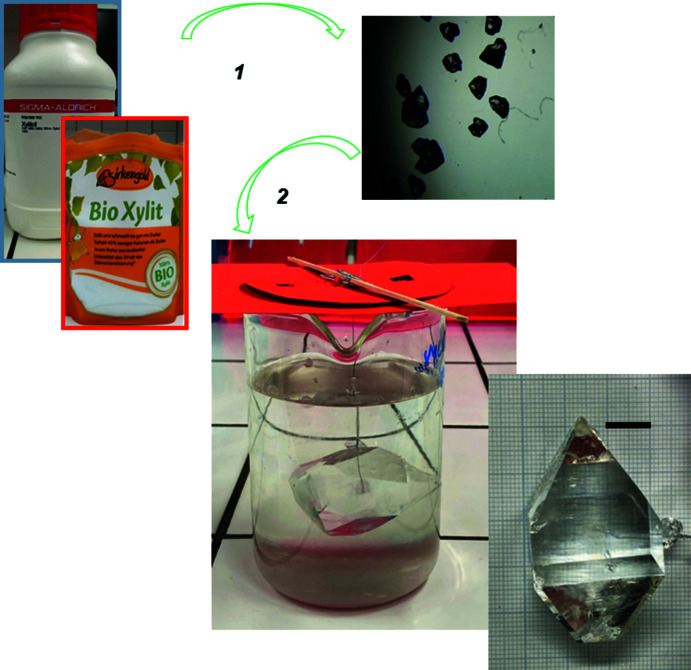
Growing large xylitol crystals. (1) Preparation of seed crystal. A seed crystal, glued to the end of a cotton thread, is placed in an aqueous saturated xylitol solution (∼170 g of xylitol dissolved in 100 mL of water). (2) After several weeks, a large single crystal is obtained. The scale bar represents 10 mm.

The first step in the process is to obtain a small seed crystal having a high degree of perfection. A supersaturated solution of each powder can be prepared by dissolving ∼170 g of xylitol or ∼60 g of erythritol in 100 mL of water heated to 60 °C. The warm solution is poured into a shallow dish and allowed to cool to room temperature. Numerous seed crystals are formed after a few hours (Fig. 4 ▸, step 1).

In the second step, one of the crystals is used as a seed crystal around which a larger crystal will grow. The seed crystal, a few millimeters in size, is attached to a cotton thread or a piece of fishing line with superglue. This step requires a little patience and dexterity; using a binocular or magnifying glass can be helpful. Securing the seed crystal with a knot is also a possibility. Once the seed crystal is attached, it is suspended in the center of an aqueous saturated solution of the sweetener using a small wooden rod or satay stick (Fig. 4 ▸, step 2). This solution is obtained as described above. It is important to wait for the solution to cool to room temperature before hanging the seed crystal in it. However, we have often observed the onset of crystallization during cooling (spont­aneous appearance of new small crystals on the walls and/or bottom of the container). Although this is not optimal as a source of unwanted crystal growth sites, it has not always been possible to avoid this phenomenon. Then cover the container with plastic wrap, aluminium foil or a piece of cardboard to keep out dust and to reduce temperature fluctuations. As solubility is quite sensitive to temperature, it is a good idea to place the set-up inside a Styrofoam box or picnic cooler.

**Figure 5:**
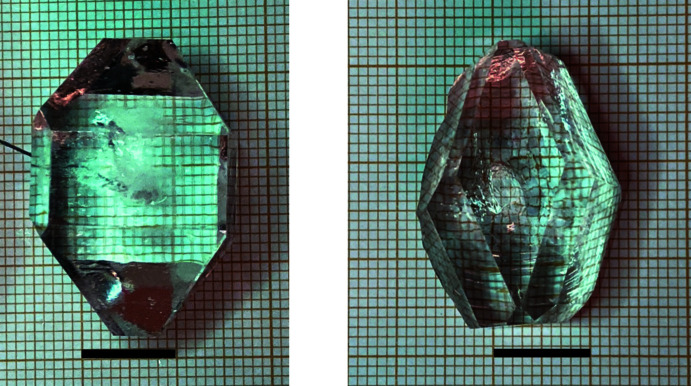
Large single crystals of xylitol (left) and erythritol (right). The scale bar represents 10 mm.

Obtaining a large crystal (a few centimeters; Fig. 5 ▸) can take some time (several weeks or months). For this, the solution must be kept sufficiently saturated otherwise the crystal growth will stop. A *general procedure* is to take the crystal out of the solution, dip it dry, and remove eventually any small crystals on the surface. Next determine the weight of the crystal and compare it to the previous one. Re-saturate the solution by adding the amount of material that had crystallized out. Warm and stir the solution until everything is dissolved. Cool the solution to room temperature and resuspend the crystal back into the newly saturated solution. Repeat the previous steps as needed. However, *another (simpler) approach* can also be successful. Keep an extra stock of saturated solution in a closed bottle. Depending on the solution level (or degree of evaporation), carefully add additional saturated solution to replace the amount of solvent that has evaporated.

## Determination of the heat of dissolution

The two selected sweeteners, erythritol and xylitol, are characterized by an endothermic heat of dissolution. This property is the basis of the fresh ‘mouthfeel’ of both substances. This effect is particularly pronounced for xylitol, which is used as a sweetener in ‘breath-freshening’ products.

The heat of dissolution (enthalpy change on dissolution, Δ*H*_diss_) of a solute can be determined by calorimetry. This method is based on the measurement of a temperature change (Δ*T*) caused by the addition of a certain amount of compound to be dissolved (*m* = mass of solute) to a certain amount of water (*m*_H2O_ = mass of water). In the case of an endothermic phenomenon, the dissolution is accompanied by a drop in the temperature of the solution. The amount of heat (*Q*) required for dissolution is proportional to the temperature drop and depends on the amounts of solute and solvent (water) and on the heat capacity *C* (or specific heat *c*) of the solvent (*c*_H2O_ = 4185 J kg^−1^ K^−1^). In the absence of solute dissociation, which is the case with the polyols studied, the change in the dissolution enthalpy Δ*H*_diss_, except for the sign, is equal to the amount of heat associated with dissolving one mole of solute (*n* = *m*/*M*, where *M* is the molar mass of the solute) [Equation (1)].

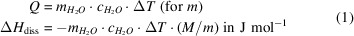




**Figure 6:**
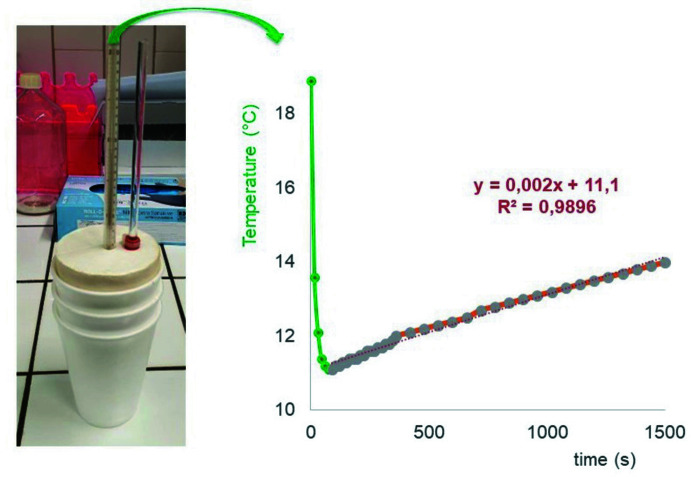
Determination of the heat of dissolution (Δ*H*_diss_) of erythritol using a simple but effective calorimetry device. The evolution of the temperature of the erythritol solution over time makes it possible to estimate, by extrapolation, the value of the temperature change Δ*T* and Δ*H*_diss_. The endothermic nature of the dissolution of erythritol is clearly demonstrated by the rapid temperature drop of the solution.

A simple setup – the so-called *coffee cup calorimeter* – was used to easily determine the heat of dissolution (Δ*H*_diss_) of erythritol and xylitol in water at school (or even at home). The set-up (Fig. 6 ▸) consists of an insulated vessel (three polystyrene cups nested together) into which 60.0 g of water is brought to room temperature (18.9°C in the example) for the erythritol experiment. After addition of 12.5 g (0.102 mol) of erythritol, the beaker is closed with a lid fitted with a thermometer and a glass tube to homogenize the solution. A rapid temperature drop is observed in the first seconds of dissolution, indicating the highly endothermic nature (Δ*H*_diss_ > 0) of the dissolution of the polyol. The minimum temperature is derived from the fitted straight line corresponding to the temperature increase associated with the gradual heating of the solution. Extrapolation to the initial time gives a temperature of 11.1°C, or a temperature change Δ*T* = −7.8°C (= −7.8 K). The heat of dissolution of erythritol is then calculated as: 

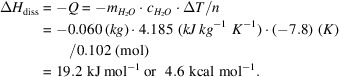




Although slightly lower than the literature value of 5.57 ± 0.01 kcal mol^−1^ at 25°C (Parks & Manchester, 1952 ▸), this estimate of the heat of dissolution Δ*H*_diss_ of erythritol seems acceptable in the context of the set-up used.

## Additional graphical tools

Crystallography is the method of choice to determine the mol­ecular structure of crystalline compounds. The X-ray diffraction technique applied to a single crystal leads to three-dimensional (3D) structures that are collected in databases such as the Cambridge Structural Database (CSD; Groom *et al.*, 2016 ▸). Some of this data (CSD Teaching Subset) is available for free (see https://www.ccdc.cam.ac.uk/Community/educationalresources/teaching-database). This is especially the case for the coordinates of the 3D structures of xylitol (refcode XYLTOL01; Madsen *et al.*, 2003 ▸) and erythritol (refcode MERYOL03; Ceccarelli *et al.*, 1980 ▸). These data are also available *via* https://www.ccdc.cam.ac.uk/structures/Search? with the option ‘Simple Search’ and using the refcode of the structure as identifier.

**Figure 7:**
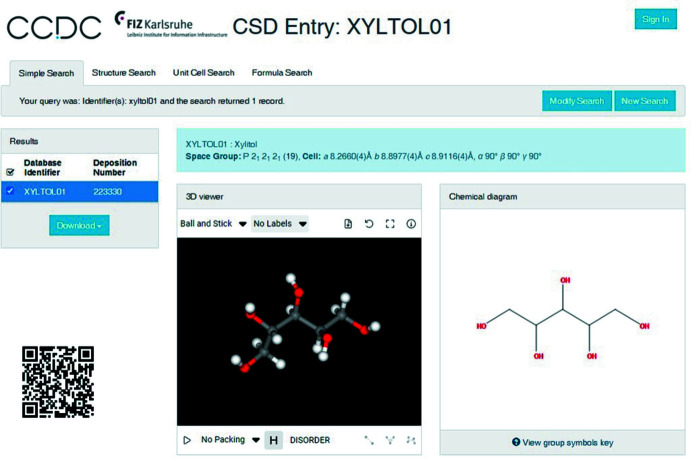
WebCSD graphical web inter­face illustrating the inter­active visualization of one of the crystal structures of xylitol (refcode XYLTOL01). The QR code enables quick access.

In addition to a web-based graphical inter­face, WebCSD (Fig. 7 ▸) that allows inter­active visualization of crystal structures, it is also possible to install the free *Mercury* software (Macrae *et al.*, 2020 ▸; https://www.ccdc.cam.ac.uk/support-and-resources/Downloads/) that allows visualization and analysis of crystal structures. These graphical tools provide access to the conformation of individual mol­ecules, as well as to the unit cell and the packing of the mol­ecules in the unit cell. The unit cell corresponds to the elementary building block, from which the entire crystal is generated by translation in three directions. Within the unit cell, the entities (ions, atoms, mol­ecules) are arranged in such a way as to maximize the stabilizing inter­actions and the packing efficiency. Symmetry elements connect the entities in the unit cell and can be used to describe the symmetry of the crystal.

The *Mercury* software is further used to illustrate the stereochemistry of the studied polyols as well as to analyze a range of crystallographic features. The visualization of these 3D structures provides a better spatial understanding; an essential skill in chemistry that needs to be developed. This approach can be used to illustrate concepts of conformation and absolute configuration of organic mol­ecules. In the case of erythritol, the graphical tool can also be used to illustrate the concept of a *meso* compound (refcode MERYOL03; Ceccarelli *et al.*, 1980 ▸) by comparison with the stereoisomers l-threitol (refcode LEDPUF; Kopf *et al.*, 1993 ▸) and d-threitol (refcode PAGDEG; Jeffrey & Huang, 1992 ▸) (Fig. 7 ▸). The spatial representation of the crystal structures allows the observed configuration to be reconciled with 2D representations for each stereoisomer. The ability to rotate the mol­ecules using graphics programs is a valuable aid in this regard, especially when no real 3D mol­ecular models are available.

**Figure 8:**
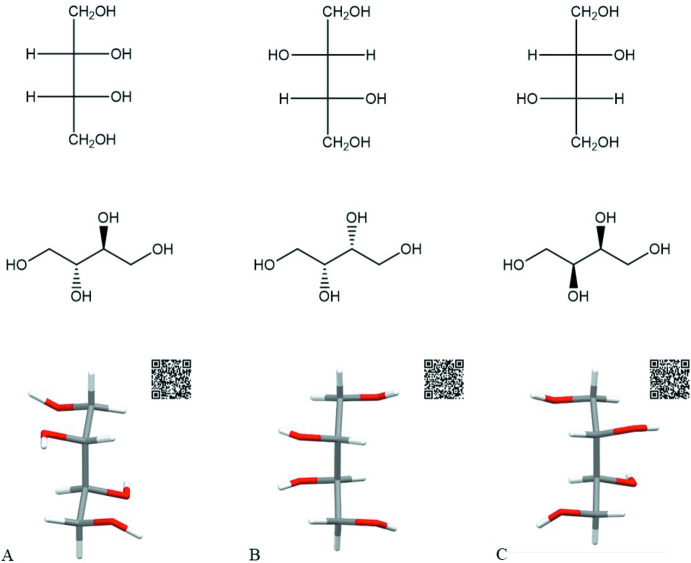
Illustration of the isomers of butane-1,2,3,4-tetrol using the 2D Fisher projection (top), structural formula (middle) and 3D structure (bottom) for *meso*-erythritol (A), d-threitol [(2*R*,3*R*)-butane-1,2,3,4-tetrol] (B) and l-threitol [(2*S*,3*S*)-butane-1,2,3,4-tetrol] (C). The 3D structure is based on the crystal structures of *meso*-erythritol (MERYOL03), d-threitol (PAGDEG) and l-threitol (LEDPUF). QR codes (also given in the supporting information) can be used for WebCSD access.

The compound butane-1,2,3,4-tetrol should have four stereoisomers because it has two stereocenters (asymmetric carbon atoms). Fig. 8 ▸ illustrates that d-threitol (B) is the mirror image of l-threitol (C). The two structures cannot be superimposed and are optically active compounds. *meso*-Erythritol (A) is optically inactive even though it contains stereocenters. Both stereocenters rotate plane-polarized light to the same degree but in opposite directions, which results in an inter­nal cancellation of optical activity. *meso*-Erythritol is superimposable with its mirror image. The 2*R*,3*S* and 2*S*,3*R* isomers are equivalent and hence only three stereoisomers are relevant. The Fisher projection of *meso*-erythritol clearly shows the presence of an inter­nal plane of symmetry through the center of the mol­ecule.

**Figure 9:**
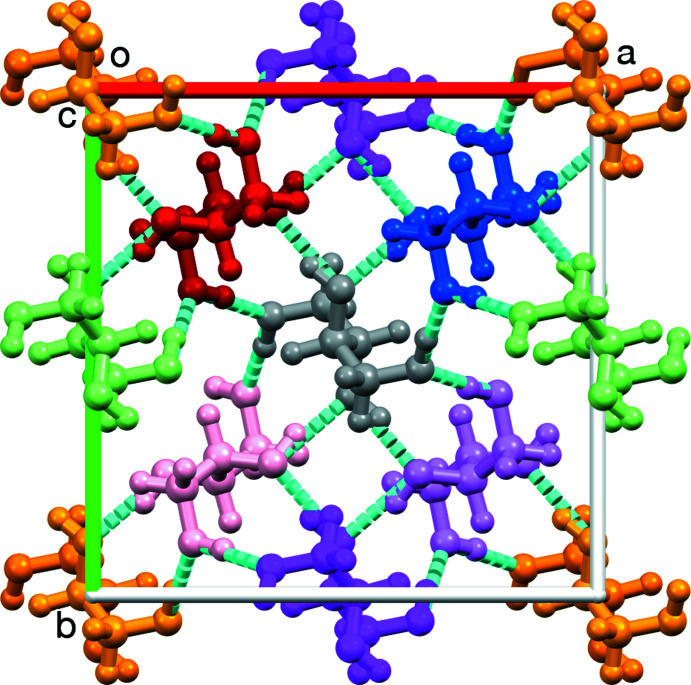
Crystal packing of erythritol viewed along the *c* axis. O—H⋯O hydrogen bonds are shown as blue dashed lines [generated with *Mercury* (Macrae *et al.*, 2020 ▸)].

The graphical tool *Mercury* also allows the illustration of other concepts such as the crystal packing, symmetry elements, Bravais lattice or space group. Inter­actions between mol­ecules in the crystal packing such as hydrogen bonds can be explored. Furthermore, high-resolution images and files to 3D print structures can be created. Fig. 9 ▸ shows the crystal packing of erythritol mol­ecules. The tetra­gonal unit cell (*a* = *b* = 12.712 Å, *c* = 6.747 Å, α = β = γ = 90°) contains eight mol­ecules, which are linked by O—H⋯O hydrogen bonds. At the vertices and in the center of the unit cell there is an erythritol mol­ecule (colored orange and gray), giving rise to a body-centered tetra­gonal Bravais lattice. The four mol­ecules colored red, green, pink and gray are related to each other by a fourfold screw axis 4_1_. The combination with the other symmetry elements present (inversion center, fourfold roto­inversion axis and glide planes) results in space group *I*4_1_/*a*.

## Conclusions

The proposed activities are based on two readily available commercial sweeteners: erythritol and xylitol. The crystallization of these compounds makes it possible, with a little patience, to obtain beautiful crystals, illustrating the principles of crystallization by evaporation through an accessible experiment. A simple calorimetric approach is proposed to demonstrate the endothermic nature of the dissolution of the selected sweeteners. This property is related to the ‘mouth-freshening’ nature of sweeteners and allows a more qu­anti­tative approach to thermochemical concepts that are sometimes abstract or difficult to illustrate. Finally, a more detailed analysis of the crystallographic structures, using freely available graphical software, allows the user to deepen further concepts of stereochemistry by providing an easy way to visualize the spatial structure of the sweeteners.

## Supplementary Material

qr code for D-threitol. DOI: 10.1107/S2056989022007617/pk2668sup1.png

qr code for L-threitol. DOI: 10.1107/S2056989022007617/pk2668sup2.png

qr code for erythritol. DOI: 10.1107/S2056989022007617/pk2668sup3.png

qr code for xylitol. DOI: 10.1107/S2056989022007617/pk2668sup4.png
