# Supplementary figures and images for: Classroom experiments with artificial sweeteners: growing single crystals and simple calorimetry
Source: Acta Crystallogr E Crystallogr Commun. 2022 Aug 2;78(Pt 9):874–9. doi: 10.1107/S2056989022007617 (PMC9443796; doi:10.1107/S2056989022007617)

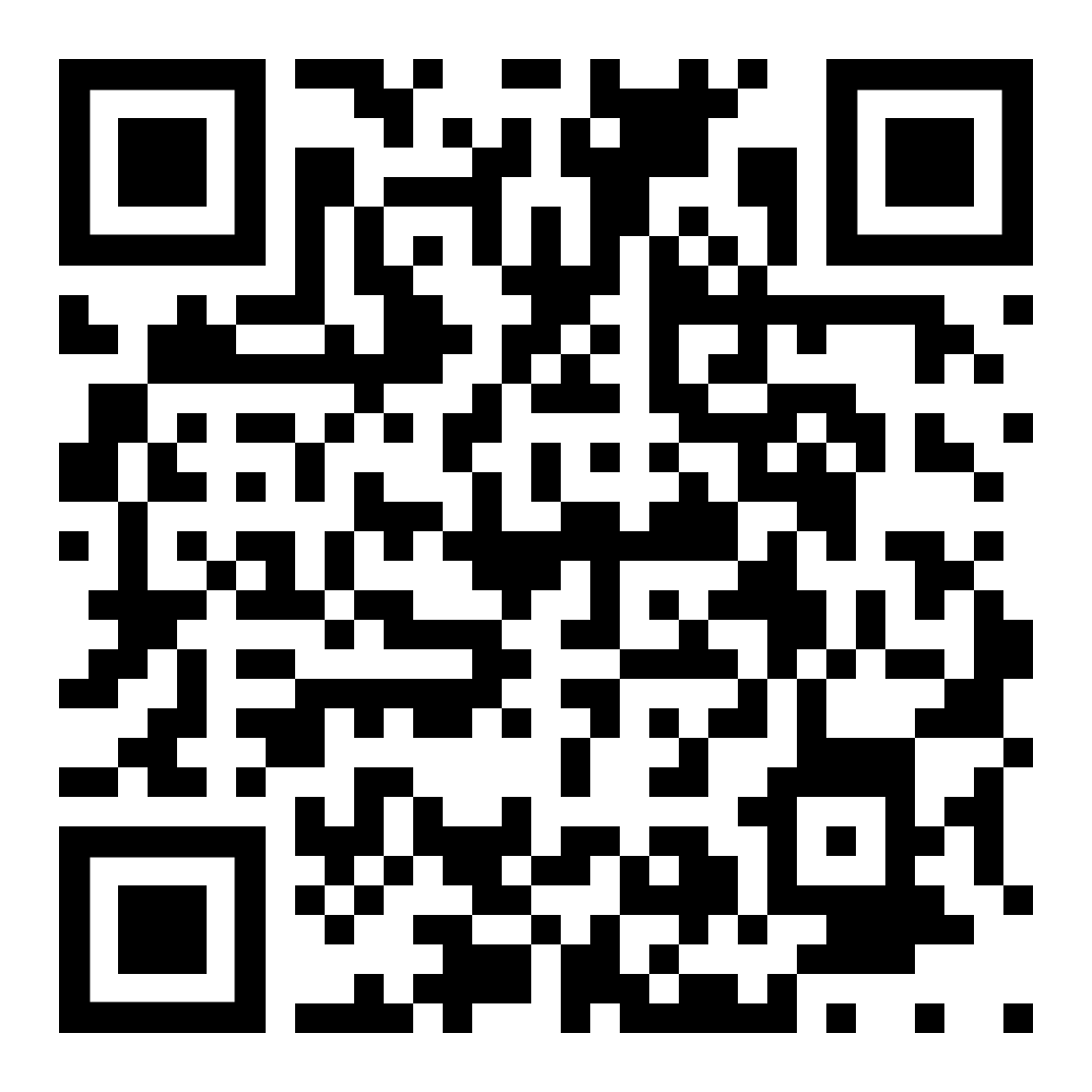

Supplement: Supplementary file 1 [file e-78-00874-sup1.png]

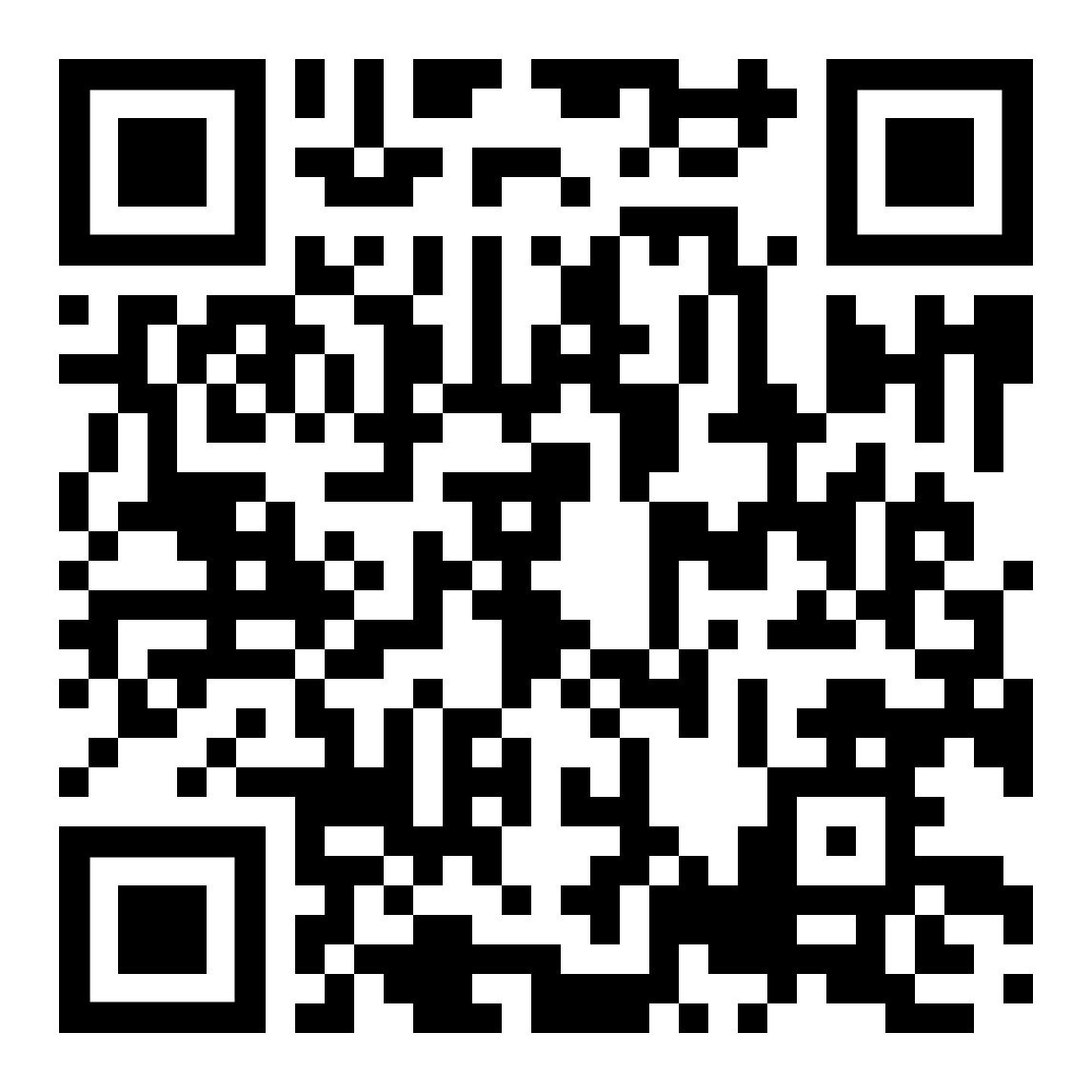

Supplement: Supplementary file 2 [file e-78-00874-sup2.png]

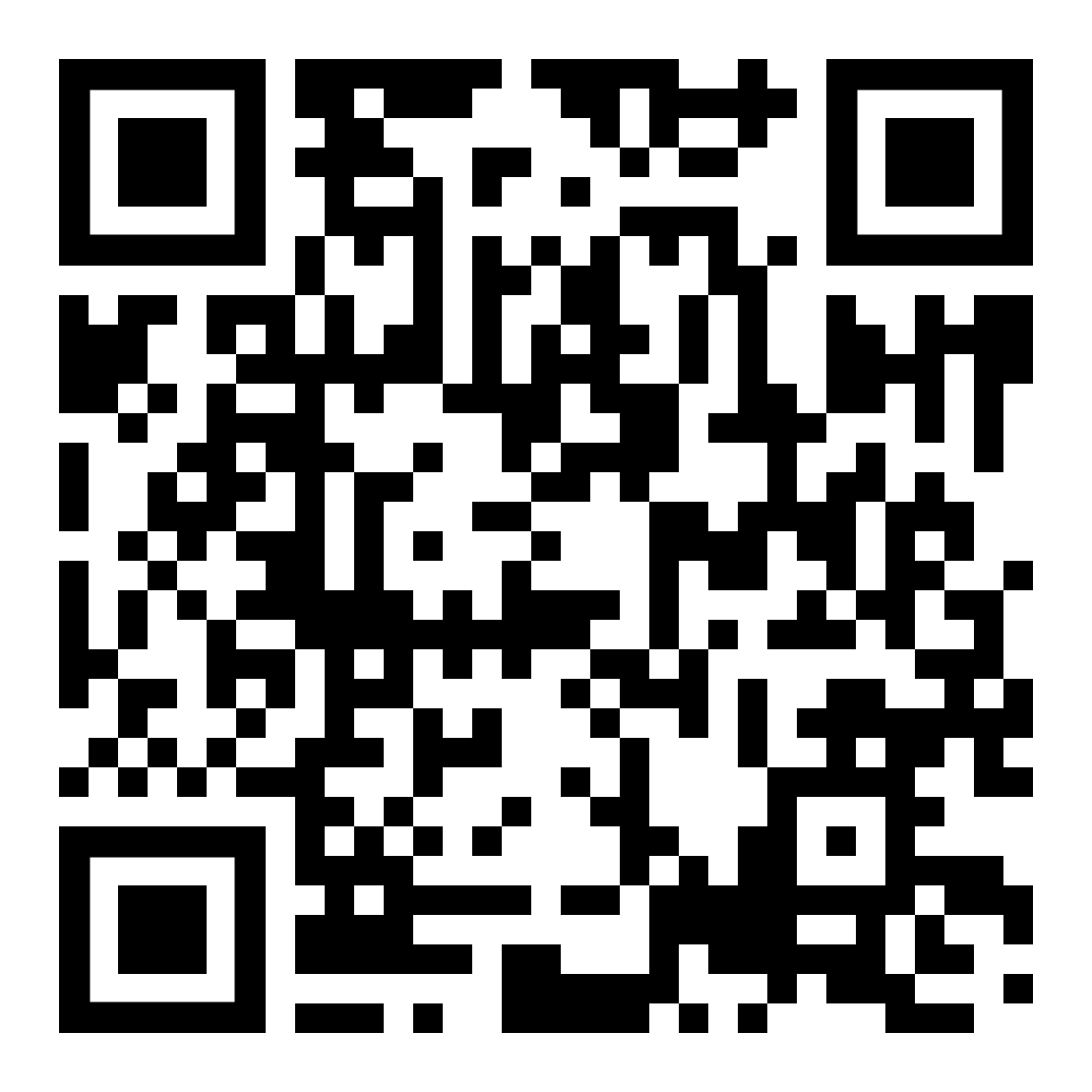

Supplement: Supplementary file 3 [file e-78-00874-sup3.png]

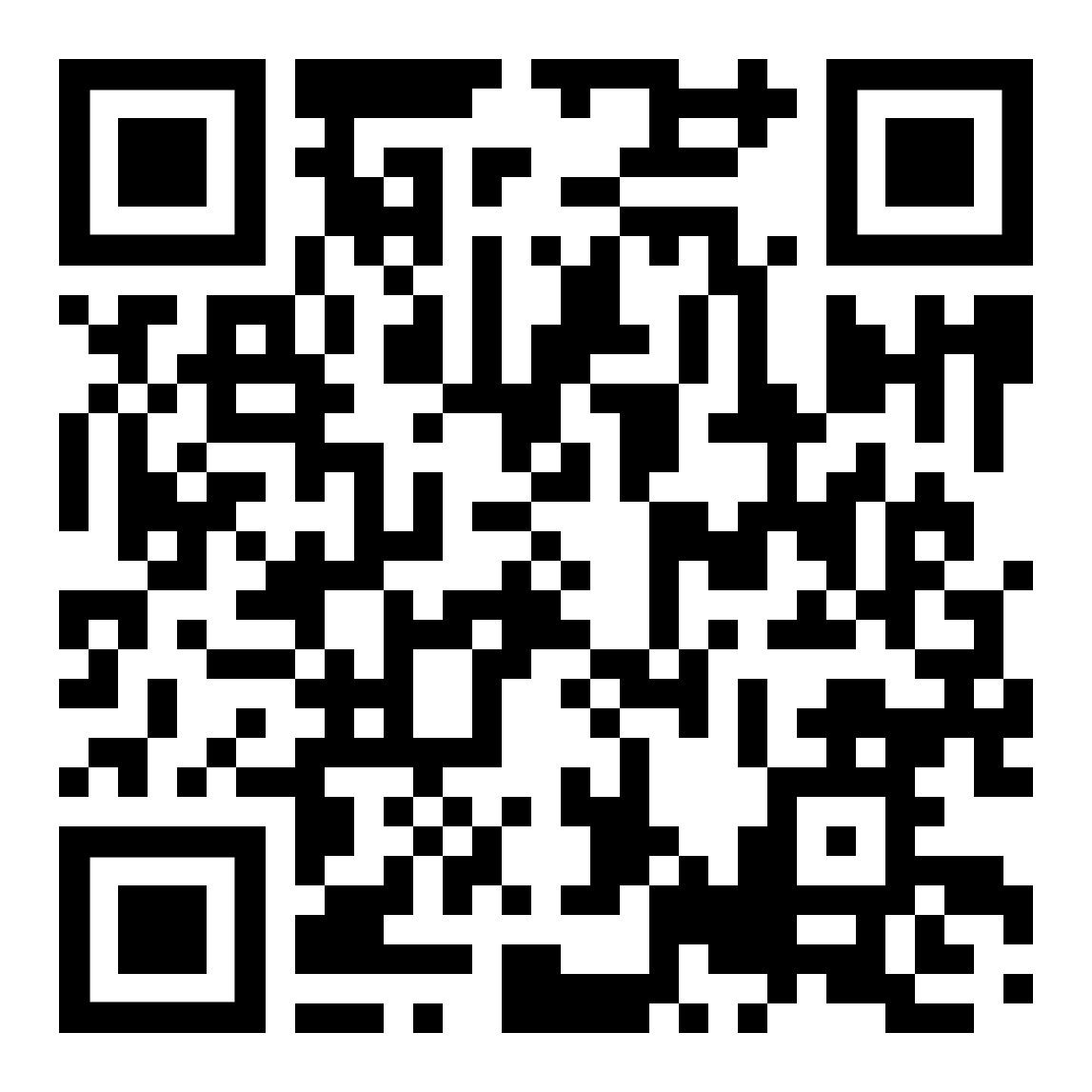

Supplement: Supplementary file 4 [file e-78-00874-sup4.png]
